# The Impact of Conservative Surgical Treatment of Adenomyosis on Fertility and Perinatal Outcomes

**DOI:** 10.3390/jcm13092531

**Published:** 2024-04-25

**Authors:** Gaby Moawad, Youssef Youssef, Arrigo Fruscalzo, Hani Faysal, Manuel Merida, Paul Pirtea, Benedetta Guani, Jean Marc Ayoubi, Anis Feki

**Affiliations:** 1 Department of Obstetrics and Gynecology, The George Washington University Hospital, Washington, DC 20037, USA; 2 The Center for Endometriosis and Advanced Pelvic Surgery, Washington, DC 22101, USA; 3 Division of Minimally Invasive Gynecology, Department of Obstetrics and Gynecology, Maimonides Medical Center, Brooklyn, NY 11219, USA; 4 Department of Obstetrics and Gynecology, HFR—Hòpital Fribourgeois, Chemin des Pensionnats 2-6, 1708 Fribourg, Switzerland; 5 Department of Obstetrics and Gynecology, Indiana University, Indianapolis, IN 46204, USA; 6 Department of Obstetrics and Gynecology, Hurley Medical Center, Michigan State University, Flint, MI 48503, USA; 7 Department of Obstetrics and Gynecology and Reproductive Medicine, Faculté de Médecine Paris, Hopital Foch, 92150 Suresnes, France

**Keywords:** adenomyosis, surgery, infertility, fertility outcomes, pregnancy

## Abstract

Adenomyosis is a benign condition commonly encountered in patients with infertility. While the definitive surgical management is hysterectomy, conservative surgical management is gaining attention in patients desiring future fertility. This review explores whether the surgical treatment of adenomyosis affects fertility outcomes for patients trying to conceive. The PubMed and Medline databases were searched using the keywords: “adenomyosis”, “surgery”, “radiofrequency”, “infertility”, “pregnancy”, “sterility”, “conception”, “miscarriage”, and “endometrial receptivity”. Abstracts were screened, and relevant articles were selected for review. This review reveals that surgery appears to improve fertility outcomes with or without medical therapy; however, the risk of uterine rupture remains high and the best technique to reduce this risk is still not known. More studies are needed to formulate the best surgical approach for preserving fertility in treating adenomyosis and to establish standardized guidelines.

## 1. Introduction

Adenomyosis remains a clinically challenging issue in gynecology and its pathophysiology is poorly understood. Possible theories include metaplasia of displaced embryonic Mullerian remnants or endometrial invasion into the myometrium due to factors such as endometrial peristalsis, intrauterine infection, or surgical procedures affecting the endometrial myometrial junctional zone. More hypotheses exist; however, none can fully explain the pathological mechanisms of adenomyosis [1].

The reported prevalence of adenomyosis ranges widely from 8% to 62% based on histopathology findings after hysterectomy and often coexists with other pathologies such as fibroids or endometriosis [2]. The wide range of estimates is often linked to various factors, such as the lack of standardized histopathologic diagnostic criteria, differences in the number of histologic tissue samples examined per hysterectomy, and variations in provider awareness levels [2]. Additionally, this might not provide an accurate representation of the true prevalence of adenomyosis, since hysterectomy is typically performed on women aged 40–50 years, thereby underestimating its prevalence in younger women [3]. Adenomyosis is not only a pathology of adult life; it involves young patients and is found to be present in 5% in adolescence and up to 44% when coexisting endometriosis features are present [4].

This condition is characterized by nonspecific symptoms, including menorrhagia, pelvic pain, and dysmenorrhea, which are the most frequently reported symptoms [2]. The treatment of adenomyosis has evolved significantly over time [5]. Historically, any patient with presumed adenomyosis underwent a hysterectomy but, over time, women started delaying their first pregnancies, and adenomyosis was encountered more often in these women who still desired fertility [5]. Since hysterectomy was no longer feasible, alternative treatments were needed. Today, despite having a broad range of options to manage adenomyosis, not a single treatment is considered curative [6]. Currently, medical treatments, mainly hormonal therapy, and uterine-preserving surgical approaches are available [6]. Although both invasive and noninvasive options exist, there is still a lack of consensus regarding the treatment of adenomyosis, and no current guidelines exist. This requires treatment to be individually tailored to each patient, including fertility preservation.

Adenomyosis is known to have a negative impact on fertility and may necessitate surgical intervention when pregnancy is desired [7]. Because there is a lack of standardization of therapy combined with scarce data on fertility outcomes, the best approach for these patients remains unclear. Some evidence suggests that fertility improves when the disease is controlled [6,8]. Hormonal treatment could achieve disease control by shrinking the adenomyotic lesions [9]. Alternatively, surgical intervention physically removes the adenomyotic lesions but also compromises the integrity of normal tissue, leading to both improvement and potential detrimental effects on reproductive outcomes [10]. The objective of the present review is to provide an updated overview of the existing literature concerning fertility and perinatal outcomes after uterine-preserving surgeries performed for adenomyosis cases.

## 2. Methods

A narrative review of the existing literature regarding fertility and perinatal outcomes after conservative surgeries conducted for adenomyosis. A literature search was performed using PubMed databases. The search words used were “adenomyosis”, “surgery”, “radiofrequency”, “infertility”, “pregnancy”, “sterility”, “conception”, “miscarriage”, and “endometrial receptivity”. Boolean operators were used to combine the search words as follows: adenomyosis AND surgery AND (Infertility OR pregnancy OR miscarriage OR sterility OR conception). The search criteria included the following: articles published in the English language, publications dating from 2000 until the present, and article types including systematic reviews, meta-analyses, randomized controlled trials, and observational studies. Abstracts were screened, duplicates were removed, and relevant articles were selected. We excluded abstracts, letters, case reports, and reviews. In addition, articles that did not report the number of patients desiring pregnancy were excluded.

## 3. Results

Adenomyosis is strongly linked to infertility and can significantly impact outcomes of infertility treatment. Adenomyosis has been diagnosed as the primary cause of infertility in over 80% of patients and has been found in more than 30% of patients who experienced previous ART failures [11]. The impact of adenomyosis on fertility is believed to be due to the proinflammatory milieu resulting from platelet aggregation and hypoxia, leading to increased levels of cytokines, prostaglandins, and local estrogen synthesis [12]. Subsequently, oxytocin signaling is induced through increased estrogen receptors, and fibrosis occurs through epithelial–mesenchymal transition and fibroblast-to-myofibroblast transdifferentiation. These factors result in myometrial hyperperistalsis, increased endometrial oxidative stress, altered steroidogenesis, and a decreased number of microvilli, which impede embryo transport and create an unfavorable environment for implantation [13]. ART is unlikely to overcome these obstacles, as adenomyosis alone, without concomitant pathology (e.g., endometriosis), has no adverse effect on folliculogenesis [14].

Adenomyosis has a deleterious effect on fertility and pregnancy outcomes. In patients undergoing ART, the likelihood of getting pregnant was reduced by 31–43% and the live birth rate (LBR) was decreased by 55%, with a significant increase in miscarriage rate (OR 2.1 to 3.4) [14]. Unfortunately, despite patients’ ability to overcome these challenges and achieve pregnancy, adenomyosis continues to pose risks to pregnancy outcomes. These risks encompass an elevated likelihood of experiencing pre-eclampsia (OR 4.35–7.87), preterm delivery (OR 2.65–3.09), delivering an infant with small for gestational age (SGA) (OR 2.86–3.90), and postpartum hemorrhage (OR 2.90) [14].

Conservative surgery for adenomyosis, with or without medical therapy, has been described as a fertility-sparing treatment for patients desiring future pregnancy. Numerous studies have highlighted the impact of these conservative techniques on fertility and pregnancy outcomes. However, the scarcity of high-quality evidence, mainly comprising case series and observational studies lacking comparable well-designed counterparts, hinders the drawing of definitive conclusions. Various classification systems have been proposed to better understand the relationship between the location, extent of adenomyosis, and the presence of concomitant pathology in relation to the severity of symptoms and/or infertility [15]. However, a clear correlation of these classification systems with infertility has not yet been established. Additionally, the available data on uterus-sparing surgical techniques do not report these classifications in their published articles.

The objective of our study is to review available conservative surgeries for the treatment of adenomyosis and their impact on infertility and pregnancy in patients seeking conception after these interventions. To better understand the available data, we grouped the studies according to the classification used in the original articles, which categorizes adenomyosis as localized/focal or extensive/diffuse.

### 3.1. Fertility Outcomes after Excisional Surgeries

#### 3.1.1. Focal Adenomyosis

Focal adenomyosis has traditionally been vaguely described as a localized area of hypertrophic distorted endometrium and myometrium, typically situated within the myometrium [16]. However, more recently, more detailed descriptive classifications have been introduced, dividing focal adenomyosis into outer and inner subtypes based on junctional zone involvement. This classification is further subdivided into groups based on the size and number of foci [17,18].

The classic technique is the primary approach used for adenomyomectomy, which involves similar steps as a myomectomy but utilizes different methods for reconstructing the uterine wall. Other wall-reconstructing techniques, such as U-shaped suturing, overlapping flaps, and triple flap techniques, have also been described [16]. These modified approaches have been utilized in both focal and diffuse adenomyosis and can be performed via an open or minimally invasive approach, including conventional laparoscopy and, more recently, robotic assistance.

Several studies have published fertility outcomes after uterus-sparing surgeries using different approaches, yielding varying results, which are summarized in (Table 1).

Fedele et al. reported the first comprehensive fertility and pregnancy outcomes after excision of focal adenomyosis. Twenty-eight patients were included in the study after diagnosis with ultrasound and MRI. Eighteen patients desired pregnancy and thirteen patients had a total of 18 pregnancies with a 50% delivery rate. Concomitant pelvic pathology (endometriosis, Mullerian anomaly, and fibroids) were present in 60% of the total patients with adenomyosis and in 77% of patients with infertility or recurrent pregnancy loss [19].

Fujishita et al. presented preliminary results from a small-sample study comparing two cytoreductive laparotomy techniques: the modified H technique and the classical excisional method. Eleven patients were included, with nine having focal and two having diffuse adenomyosis. No patients were pregnant after classical excisional surgery, while 50% (two out of four) of patients were able to achieve pregnancy after the modified H technique [20].

Wang et al. compared the symptoms, recurrence, and fertility outcomes between surgery alone and surgery combined with medical treatment in a prospective study. They used ultrasound features for diagnosis and included focal cases with adenomyosis localized to one wall (anterior 17%, posterior 70%, and fundal 11.8%), without employing any specific classification system. Among the 51 patients in the surgery group, excision techniques were performed via laparoscopy or laparotomy. Out of 27 patients desiring fertility, 20 patients (74%) achieved pregnancy and 17 (63%) had successful deliveries [21].

Guy et al. compared laparoscopic surgical excision (cytoreductive) for adenomyosis to combined surgery with medical therapy (Gestrinone) and medical therapy alone. They included only infertility patients and excluded those with extensive endometriosis. There was no comment on the extent or classification of adenomyosis. Out of the 12 cases who underwent laparoscopic surgery alone, the pregnancy rate was 41.67% (5 cases), and no pregnancy outcomes were reported [22].

Kishi et al. analyzed the fertility outcomes of 104 patients after laparoscopic excisional uterine-sparing surgery for adenomyosis. Out of 102 patients desiring pregnancy, 36 patients (35.2%) achieved pregnancy. A higher clinical pregnancy rate, defined as the presence of a fetal heartbeat after 12 weeks, was noted in patients <39 years (41.3%) compared to patients ≥40 years (3.7%) after surgery. The study used diffuse or local widening of the junctional zone on T2-weighted images (>12 mm) as an inclusion criterion but did not specify a particular classification system. In an attempt to correlate the localization of pathology by dividing uterine myometrium into outer myometrium and subendometrial myometrium (JZ), which is involved in the preparation of the endometrium for implantation and uterine peristalsis, sperm transport, and hemostasis during menstruation, their data analysis was subdivided according to the presence of endometriosis, endometrioma, anterior or posterior wall involvement, changes in the junctional zone, and Revised American Fertility Society (r-AFS) score. They concluded that patients with posterior wall involvement and higher r-AFS scores have lower chances of getting pregnant, which they previously demonstrated to be associated with extrinsic endometriosis [23,24].

Tamura et al. reported one of the largest datasets on adenomyosis and fertility outcomes through a retrospective questionnaire from 190 facilities. They compared three groups (no pretreatment, medical treatment, and surgery) before infertility treatment of patients with adenomyosis. The 23 patients who received conservative surgery for focal adenomyosis had a pregnancy rate of 39% with no miscarriages [25].

Takeuchi et al. published the results of laparoscopic excision for nine cases of juvenile cystic adenomyosis (JCA). Although previously included in other meta-analyses as part of focal adenomyosis, this article was excluded from our review, as JCA is not considered part of adenomyosis pathology [26]. Historically, juvenile cystic adenomyosis was erroneously categorized as cystic adenomyosis and considered a subdivision of focal adenomyosis. However, JCA has distinct histopathological and anatomical characteristics and is now classified as accessory and cavitated uterine masses (ACUM), representing a new Müllerian anomaly [27]. ACUM diagnosis relies on specific criteria: (i) the presence of an isolated accessory cavitated mass; (ii) a normal uterus, including the endometrial lumen, Fallopian tubes, and ovaries; (iii) confirmation through surgical excision and pathological examination; (iv) an accessory cavity lined by endometrial epithelium with glands and stroma; (v) the presence of chocolate-brown-colored fluid content within the cavity; and (vi) absence of adenomyosis if the uterus has been removed, although small foci of adenomyosis may be present in the myometrium adjacent to the accessory cavity. ACUM is predominantly diagnosed in individuals under the age of 30, although cases above this age limit have been reported [27,28].

#### 3.1.2. Diffuse Adenomyosis

Conservative surgery using different techniques has also been employed for diffuse adenomyosis in combination with other procedures (Table 1).

The foremost challenge encountered during surgical excision of diffuse adenomyosis is excessive bleeding, which may necessitate immediate blood transfusion, resulting in incomplete operation and even prompt emergent hysterectomy. Kwon et al. investigated the efficacy of transient occlusion of uterine arteries (TOUA) prior to performing a classical cytoreductive surgery in 26 patients with refractory diffuse adenomyosis. Their findings demonstrated that TOUA placement was advantageous in reducing estimated blood loss (EBL) without extending operative duration, although without reported data on pregnancy outcomes [29]. Subsequently, in 2018, Kwack et al. examined pregnancy outcomes in 116 patients with diffuse adenomyosis treated using the same approach. Among the cohort, 11 patients underwent assisted reproduction, resulting in five successful conceptions. Additionally, five patients conceived naturally, yielding a total of 10 pregnancies, three of which experienced early missed abortions while seven progressed to term without complications [30]. In another investigation by Saremi et al., they assessed the pregnancy outcomes associated with the use of TOUA alongside the wedge resection technique, aimed at reducing the risk of endometrial entry during surgical interventions [31]. Within this cohort, a total of 21 pregnancies (30%) and 16 deliveries were achieved out of 70 attempted conceptions. Among the participants, 49 underwent assisted reproduction, resulting in 14 (28.5%) pregnancies, while the remaining 21 attempted natural conception resulting in 7 (33%) pregnancies. The authors concluded that this procedure is a viable option for women desiring fertility; however, a larger number of cases are needed to establish safety and risks [30,31].

Different incisional techniques have been described for cytoreductive surgical approaches. In 2004, Fujishita et al. elucidated the fertility outcomes of the H-incision technique compared to the classical approach. The H incision entails a vertical incision complemented by two horizontal incisions resembling the letter “H”, facilitating wide serosal exposure and substantial removal of adenomyotic tissue. This technique has been used in localized and generalized disease [20]. This technique demonstrated promising outcomes. Out of the 41 patients who underwent the H-incision technique, 31 expressed a desire for pregnancy. Notably, 38.7% of these patients achieved clinical pregnancy and 22.5% successfully reported live births [32]. The authors highlighted advantages of the H-incision approach that potentially contributed to successful pregnancies, including extensive removal of adenomyotic tissue, decreased tissue tension, and improved assessment of endometrial perforation [20,32].

Various resection techniques have been reported, including the utilization of argon beam laser, asymmetric resection, monopolar needle, or myolysis, among others. Yoon et al. described a novel technique with the argon laser where a T or transverse H incision was performed through laparotomy, followed by a serosal flap and subsequent shaving of the adenomyometrial tissue with the argon beam guided by ultrasound until residual myometrial thickness was at least 1 cm [33]. As previously discussed, various suturing techniques have been used to close uterine defects post-surgery to decrease the risk of myometrial gaps and uterine rupture. Options include multilayer suturing, U-shaped suturing, the overlapping flap technique, or the triple flap technique [34]. Osada et al. introduced a novel adenomyomectomy method involving radical excision of adenomyotic tissues and uterine wall reconstruction using the triple-flap method, without overlapping suture lines, to mitigate rupture risks in subsequent pregnancies [35]. Out of 26 women aspiring to conceive, 16 achieved pregnancy using this approach, with 14 delivering healthy babies (53.8%). Among these pregnancies, 4 occurred spontaneously, while the remaining 12 were achieved through assisted reproduction. Notably, the two spontaneous abortions occurred in the assisted reproduction group, with no instances of uterine rupture reported [35]. The authors concluded that the triple-flap method offered several advantages, enabling wider and more thorough excision of affected tissues than conventional wedge resection. Furthermore, it facilitated improved uterine wall reconstruction, maintaining adequate thickness with three myometrial layers, enhancing the uterus’ capacity to sustain normal pregnancies without rupture risks [35]. In the retrospective questionnaire by Tamura et al., 61 patients underwent surgery for diffuse adenomyosis, with a pregnancy rate of 39.3%, which was not inferior to that of patients who received pre-treatment medication (PR = 42.9%) [25].

**Table 1:** Studies evaluating fertility outcomes after surgery for adenomyosis.

Fertility Outcomes after Uterus Conservative Surgeries for Focal Adenomyosis								
Name of Author	Type of Study	N	Method of Diagnosis	Classification	Surgical Approach	N Desiring Fertility	N(%) Getting Pregnant/Method	Successful Deliveries
Fedele et al., 1993 [19].	Retrospective	28	US and MRI	Focal	Excision/Laparotomy	18	13 patients (72%) 18 pregnancies 17 natural, 1 IVF	9 (50%)
Fujishita et al., 2004 [20].	Retrospective	9	US ± MRI	Focal	Laparotomy: Cytoreductive 4 Excision/ 5 modified H	3 4	0 2 (50%), Natural	1 CS & 1 ongoing
Wang et al., 2009 [21].	Prospective	51	US	Focal	Excision/ Laparotomy or Laparoscopy	27	20 (74%) All Natural	17 (63%)
Kishi et al., 2014 [23].	Retrospective	104	MRI	Ant wall Post wall JZ changes Endometriosis	Excision by laparoscopy	102	Total preg. 36 (35.2%) Clinical preg. 32 (31.4%) 16 natural/16 IVF	N/A
Guy et al., 2016 [22].	Retrospective	12	US	Focal (Local Adenomyoma)	Laparoscopy Excision	12	5 (41.6%)	N/A
Tamura et al., 2017 [25].	Retrospective Questionnaire	336	US and MRI	Focal	N/A	23	9 (39%)	N/A
Fertility Outcomes after uterus conservative surgeries for Diffuse adenomyosis								
Saremi et al., 2014 [31].	Prospective	103	TVUS and HSG	Diffuse	Laparotomy Wedge technique and overlapping flaps	70	21 patients (30%) 14 ART 7 Natural	16 (22.8%)
Fujishita et al., 2010 [32].	Retrospective	41	N/A	N/A	Laparotomy H- technique	31	12 patients (38.7%)	7 (22.5%)
Osada et al., 2011 [35].	Prospective	104	MRI	Diffuse	Laparotomy Triple-flap method	26	16 patients (61.5%) 12—ART 4—Natural	14 (53.8%)
Tamura et al., 2017 [25]	Retrospective Questionnaire	336	TVUS and MRI	Diffuse	Laparotomy and laparoscopy	61	24 (39.3%)	N/A

### 3.2. Fertility Outcomes after the Combination of Surgery and Medical Therapy for Adenomyosis

Medical therapy has been employed in the treatment of adenomyosis either alone or in conjunction to surgery. Medications used in these protocols included GnRH-a most commonly, followed by combined oral contraceptives (C-OCP), gestrinone, and aromatase inhibitors [34,36]. Despite some studies proving superiority of a combined approach as opposed to a more classical operative treatment, fertility outcomes have been inconsistent across studies reporting different treatments [34,37] (Table 2).

Yoon et al. evaluated the effects of combination therapy involving GnRH-a administration for 3 cycles following surgical treatment. Of the 45 patients undergoing this treatment, 33 (54.5%) attempted to conceive. Among them, 18 (39%) became pregnant; 3 conceived naturally, while the remainder achieved pregnancy through ART [33]. Rajuddin et al. compared laparoscopic resection combined with GnRH agonist to aromatase inhibitor therapy with anastrozole for the treatment of adenomyosis. Their cohort consisted of 32 patients who underwent combined surgery/medical treatment and 23 who received medical treatment [38]. Out of 32 cases who received combined therapy, 3 achieved pregnancy resulting in 2 live births and 1 miscarriage [38]. Pregnancy rates were reported to be comparable between these two groups, with 9.4% for surgery vs. 8.6% for medical therapy at three months of medical treatment [38]. Hadisaputra et al. studied the combination of GnRH agonist administration for 3 months following either myolysis or laparoscopic excision. Out of 10 patients who underwent laparoscopic excision, 3 became pregnant and 2 of these pregnancies resulted in successful deliveries [39].

Another study by Al-jama et al. comparing medical treatment with GnRH to combination therapy revealed that medical therapy alone has a short effective period for conception, while combined surgical and medical treatment offered better pregnancy rates and a sustained ability to conceive. In this study, 13.6% of patients receiving GnRH achieved pregnancy within 18 months of stopping treatment, while 44.4% of patients undergoing combination therapy became pregnant up to 36 months post-treatment (*p* = 0.0393) [40]. Guy et al. compared the outcomes of medical, surgical, and combination treatment for adenomyosis [22]. They described three groups of patients: Group A consisted of 27 patients who underwent laparoscopic cytoreductive surgery and received gestrinone; Group B consisted of 25 patients who only underwent laparoscopic surgery; and Group C had 30 patients who only received gestrinone [22]. Pregnancy rates were only significantly different between the combination treatment and medical therapy alone (57.14% vs. 15.38%, *p* = 0.06). No significant differences were seen when comparing Group A to B (57.14% vs. 41.67%, *p* = 0.695) or Group B to C (*p* > 0.05). The authors concluded that surgery may be beneficial when the goal is to preserve fertility [22].

Chang et al. conducted a study to determine the factors influencing treatment success in combination therapy. They discovered that younger age, lower baseline analgesic-use score, BMI, and anterior location of the adenomyoma were associated with live births. The authors advised caution when using this approach in older patients seeking fertility [41]. A lower level of CA-125 (<15 IU/mL) was also shown to be a significant predictor of spontaneous pregnancy in a small series of patients undergoing conservative surgery and GnRH agonist treatment for adenomyosis [42]. Huang et al. published a case series involving nine patients with unexplained infertility. Of these, three patients became spontaneously pregnant, while the remaining six were unsuccessful in conceiving, even after multiple IVF attempts. Interestingly, they reported lower CA 125 levels (less than 15 IU/mL) in the patients who became pregnant. Based on these findings, they suggested that CA 125 levels might be a reliable indicator for monitoring the efficacy of conservative surgery combined with other treatments [42]. Al Jama et al. also demonstrated that a larger decrease in the CA-125 levels (*p* = 0.0009) was shown to be a significant predictor of clinical pregnancy in patients receiving a combination of surgery and GnRH agonist [40]. Thus, we can infer that lower preoperative and postoperative levels of CA-125 are associated with improved outcomes [40,41,42].

Zhou et al. reported the outcomes of a combined double flap adenomyomectomy for diffuse adenomyosis, followed by postoperative GnRH agonist treatment for 3–6 cycles. Among the 137 patients who desired pregnancy, 62 were able to conceive (56.5% spontaneously and 43.4% through IVF). They concluded that the cumulative pregnancy rate (CPR) within the first three years was as high as 70.1% when the postoperative junctional zone maximum-A (JZmax-A) measured ≤8.5 mm [43]. Wang et al. compared the reproductive outcomes of combination therapy vs. surgery alone; they showed higher clinical pregnancy rate in combination, although not statically significant (79% vs. 74%), but combination therapy was superior in the successful delivery rate (73% vs. 63%) with a higher proportion of term deliveries (61% vs. 56%) [21,44].

**Table 2:** Studies evaluating fertility outcomes after the combination of surgery and medical therapy for adenomyosis.

Fertility Outcomes after Surgery Combined with Medication							
Name of Author	Type of Study	N	Natural vs. ART	Medication Used Combined with Surgery	N Desiring Fertility	(%) Getting Pregnant	Successful Deliveries
Yoon et al., 2023 [33]	Prospective trial	50	ART	GnRH-a: 3 cycles	33	54.5%	30.3%
Rajuddin et al., 2004 [38]	Retrospective study	32	Natural	GnRH-a: 3.6 mg for 4 weeks, then twice every 4 weeks	32	9.4%	6.3%
Hadisaputra et al., 2006 [39].	Randomized control trial	20	Natural	GnRH-a:3 cycles	10	30%	20%
Al-Jama et al., 2011 [40].	Retrospective study	18	Natural	GnRH-a: 3.5 mg every month for 6 months	18	44.4%	33.3%
Guy et al., 2016 [22].	Retrospective study	27	Natural	Gestrinone: 2.5 mg twice a week for 3 months	14	57.14%	-
Chang et al., 2013 [41].	Retrospective study	56	Natural	GnRH-a: 6 times a month	56	41.1%	26.8%
Huang et al., 2011 [42].	Retrospective study	9	Both	GnRH-a: 3.6 mg for 6 cycles	9	33.3%	22.2%
Zhou et al., 2022 [43].	Retrospective study	137	Both	GnRH-a: 3–6 courses	137	40.9%	32.8%
Wang et al., 2009 [21].	Prospective non-randomized study	114	Natural	GnRH-a: 6 courses	44	79.5%	72.7%
Wang et al., 2009 [44].	Prospective study	28	Natural	GnRH-a: 3.75 mg every 4 weeks for 24 weeks	28	46.4%	32.1%

### 3.3. Perinatal Outcomes

Adenomyosis is widely recognized for its correlation with various adverse pregnancy outcomes, including uterine rupture, abnormal placentation, preterm labor, premature rupture of membranes, and fetal growth restriction [45] (Table 3). Hashimoto et al. highlighted these associations in a retrospective case–control study, revealing significant links between adenomyosis and second-trimester losses (OR: 11.2), pre-eclampsia (OR: 21), placental malposition (OR: 4.9), and preterm delivery (OR: 3.1) compared to controls [45].

Several studies have documented adverse pregnancy outcomes linked to uterine-sparing surgical management of adenomyosis. Placenta accreta spectrum and uterine rupture pose significant morbidity and mortality risks. These complications may stem from adenomyosis-induced alterations in myometrial strength and perfusion, compounded by surgical trauma that may affect uterine healing [46]. Kwack et al. documented four cases of abnormally adhered placentas among 22 adenomyomectomy cases and one case of uterine rupture in a patient with a history of high-intensity focused ultrasound and multiple prior adenomyomectomies before conception; although, they did not stratify such complications with the type of adenomyosis resected [46]. Similarly, Sayama et al. observed a significant increase in placenta accreta spectrum (*p* < 0.01) in patients with focal adenomyosis who underwent complete resection (9/18) compared to those who did not (0/105); they reported a case of uterine rupture that required cesarean hysterectomy for bleeding control in one of the patients with abnormal adhered placenta [47].

It is well known that, in patients with a history of uterine surgeries, there is a higher likelihood of uterine rupture, a risk that increases when entry to the endometrial cavity happens during surgery or when preterm labor is present. Sugiyama et al. conducted a retrospective cohort study monitoring pregnant women who underwent adenomyomectomy of focal adenomyosis, observing that, out of 10 patients, 3 required emergency cesarean section due to tocolytic-resistant spontaneous preterm labor [48]. Comparing these cases with those undergoing elective cesarean section revealed shorter cervical lengths upon admission and a significantly higher rate of cervical length shortening. While no uterine ruptures were reported, patients undergoing emergency cesarean section exhibited significant myometrial windows at delivery [48]. Another study investigated uterine wall thickness in patients undergoing cytoreductive surgery for diffuse adenomyosis and its association with uterine rupture [49]. Preconception and early pregnancy diagnostic imaging revealed that patients with uterine wall thickness <7 mm had an increased risk of uterine rupture (two out of five patients), although sensitivity for predicting rupture was 0%. The study suggested that a minimum wall thickness of 9–15 mm is optimal for conception and preventing uterine rupture during pregnancy, while <7 mm may pose an elevated risk of subsequent rupture [49].

Ono et al. conducted a retrospective cohort study assessing the impact of laparoscopic conservative surgery on perinatal outcomes, including preterm delivery, hypertension, diabetes in pregnancy, fetal growth restriction, abnormal placentation, uterine rupture, cesarean delivery rate, NICU admission, and neonatal deaths, among others [50]. The study revealed a lower prevalence of fetal growth restriction in the surgical group and an increased rate of cesarean section, with no significant differences observed in other obstetrical complications [50]. These findings are consistent with those of Sayama et al., demonstrating that focal adenomyomectomy may decrease the incidence of prelabor premature rupture of membranes (*p* > 0.05), pre-eclampsia (*p* > 0.05), and small for gestational age (*p* < 0.05) [47]. Their conclusion suggests that, while the primary aim of adenomyomectomy is complete lesion resection, prioritizing the preservation of the normal uterus to avoid endometrial failure may hold greater significance from a perinatal prognosis standpoint. Hadisaputra et al. compared myolysis with sharp bipolar instruments vs. resection with a monopolar needle. They reported 3 pregnancies out of 20 patients in the resection group and 2 pregnancies out of 10 patients in the myolysis group [39]. Early membrane rupture occurred in one patient in the resection group and uterine rupture occurred in one patient in the myolysis group. Even though fertility outcomes were not the main variables investigated in the study and the cohort was too small to draw conclusions, the authors recommended against myolysis for patients with adenomyosis with a desire for pregnancy [39].

**Table 3:** Studies evaluating pregnancy outcomes after conservative surgery for adenomyosis.

Pregnancy Outcomes after Uterus Conservative Surgeries for Adenomyosis						
Name of Author	Type of Study	N	Classification	Surgical Technique	N of Clinical Pregnancies (Deliveries)	Pregnancy Outcomes
Kwack et al., 2021 [46]	Retrospective	466	- Focal and Diffuse	- Laparotomy with classical technique, and medical	(22)	8 Preterm deliveries 2 Placenta accreta & 2 previa 1 Uterine rupture 8 NICU admission
Sayama et al., 2023 [47]	Retrospective	123	- Focal	- Laparotomy with asymmetrical resection or triple-flap method (18), and expectant management (105)	18 (18)	- 9 Placenta accreta - 1 Preterm labor - 1 Uterine Rupture - Significant decrease in PROM, Preeclampsia, SGA
Sugiyama et al., 2020 [48]	Retrospective	10	- Focal and Diffuse	- Laparotomy	10 (10)	- 3 Preterm labor
Otsubo et al., 2016 [49]	Retrospective	23	- Diffuse	- Laparotomy, asymmetrical technique	23 (13)	- 8 Miscarriages
Ono et al., 2023 [50]	Retrospective	43	- Focal and Diffuse	- Laparoscopy	17 (17)	- 5 Preterm deliveries
Wang et al., 2009 [44]	Retrospective	165	- Focal	- Laparotomy or laparoscopic, and medical	55 (49)	- 6 Miscarriages - 7 Preterm deliveries
Kishi, et al., 2014 [23]	Retrospective	102	- Diffuse	- Laparoscopy	32 (15)	- 2 Placenta accreta
Tamura et al., 2017 [25]	Retrospective	84	- Focal and Diffuse	- Laparotomy and laparoscopy	33 (-)	- 10 Miscarriages
Saremi et al., 2014 [31]	Prospective	103	- Diffuse	- Laparotomy with wedge technique with overlapping flaps	21 (16)	- 4 Miscarriages - 2 Uterine ruptures - 1 Stillbirth
Yoon et al., 2023 [33]	Retrospective	50	- Diffuse	- Laparotomy with Argon beam, and medical	18 (10)	- 8 Miscarriages
Osada et al., 2011 [35]	Prospective	104	- Diffuse	- Laparotomy with triple-flap method	16 (14)	- 2 Miscarriages
Nishida et al., 2009 [51]	Retrospective	44	- Diffuse	- Laparotomy with asymmetric resection	2 (1)	- 1 Interstitial pregnancy
Hadisaputra et al., 2006 [39]	Randomized control trial	20	- Focal and Diffuse	- Laparoscopic with or without myolysis, and medical	5 (2)	- 1 PROM - 1 Uterine rupture - 1 Stillbirth
Rajuddin et al., 2006 [38]	Retrospective	32	-	- Combined surgical and medical	3 (2)	- 1 Miscarriage
Al-Jama et al., 2011 [40]	Retrospective	18	- Focal	- Classical technique, and medical	8 (6)	- 2 Miscarriages
Chang et al., 2013 [41]	Retrospective	56	- Focal	- Laparotomy and medical	27 (15)	- 4 Miscarriages - 1 Ectopic pregnancy - 2 Preterm deliveries
Zhou et al., 2022 [43]	Retrospective	137	- Diffuse	- Laparotomy and medical	62 (45)	- 14 Miscarriages - 6 Preterm deliveries

### 3.4. Systematic Reviews and Meta-Analysis of Fertility Outcomes after Adenomyosis Uterus-Sparing Surgery

Few meta-analyses have focused on reviewing data regarding fertility and pregnancy outcomes after uterus-sparing surgeries specifically for patients wishing to conceive after surgery, yielding inconsistent results (Table 4).

Interestingly, the analysis reported comparable PRs can be achieved through both natural conception and assisted reproductive technology (ART) after fertility-conserving surgery for focal and diffuse AD [34].

Rocha et al. conducted an analysis encompassing 16 articles to assess the reproductive outcomes of conservative treatments for patients with adenomyosis-associated infertility. Among the six studies focusing on surgical treatment, the overall pooled clinical pregnancy rate after surgical resection of adenomyosis was determined to be 38.8%, with a range from 12.5% to 61.5%. Notably, the pooled miscarriage rate was 17.9%, while the pooled live birth rate was 30.4%. However, when considering only spontaneous pregnancies, the overall clinical pregnancy rate was notably low, at 18.2%. Additionally, when gonadotropin-releasing hormone agonists (GnRH-a) were utilized for 24 weeks post-surgery, the pooled spontaneous pregnancy rate was significantly higher compared to not using adjuvant GnRH-a (40.7% vs. 15.0%). Interestingly, there was no significant difference observed between the pooled results with or without GnRH-a post-adenomyomectomy concerning pregnancy rate, live birth rate, in vitro fertilization (IVF) pregnancy rate, or miscarriage rate. Moreover, among the 10 studies focusing on assisted reproductive technology (ART), an overall clinical pregnancy rate of 36.1%, an overall miscarriage rate of 25.9%, and an overall live birth/ongoing pregnancy rate of 29.9% were reported. Furthermore, upon comparing the long and short stimulation protocols of ART in patients with adenomyosis and infertility, a higher pooled clinical pregnancy rate (43.3% vs. 31.8%, respectively), a higher live birth/ongoing pregnancy rate (43% vs. 23.1%), and a lower frequency of miscarriage (18.5% vs. 31.1%) were observed. These findings underscore the complexity of adenomyosis-related infertility and emphasize the importance of tailored treatment approaches to optimize reproductive outcomes [37].

A recent meta-analysis, including nine excisional surgical studies and four non-excisional studies (HIFU, MWA, RFA, and UAE), concluded that there were no statistical differences in the pregnancy rates between the excisional group (40%) and the non-excisional group (51%), albeit with high heterogeneity in the excisional group [52]. Additionally, there was no statistical difference, with no heterogeneity observed, in the rates of miscarriage (21% vs. 22%) or live births (70% vs. 71%) between the excisional and non-excisional groups simultaneously. A subgroup analysis was performed to identify the source of heterogeneity, which revealed that the type of adenomyosis (focal vs. diffuse) and method of conception (natural vs. ART) can be sources of heterogeneity, rather than the study design (cohort study vs. case series) or the intervention model (combined surgical–medical treatment vs. treatment alone). Furthermore, the pregnancy rates of the excisional group were higher via ART (41%) compared to the natural approach (28%). However, there was no difference in pregnancy outcomes between diffuse or focal cases, which contradicts Tan et al.’s findings that showed better pregnancy results for focal excisional surgery compared to diffuse cases [34,52].

Another systematic analysis by Grimbizis et al. analyzed the fertility outcomes after uterus-sparing techniques by grouping data according to the extent of adenomyosis tissue and healthy myometrium removed during surgery [16]. The proposed categories included:

(1) Complete excision of adenomyosis, which ensures the complete removal of all clinically recognizable non-microscopic lesions while maintaining the integrity of the uterine wall. This can be achieved by either performing adenomyomectomy, which can be used in cases of localized adenomyosis (adenomyoma) but also in selected cases of more diffuse adenomyosis with reconstruction of the uterine wall, or cystectomy, used in cases of cystic focal adenomyosis, including the entire removal of the adenomyotic cyst. This can be achieved by classic excisional techniques or modifications in wall reconstructions using U-shaped suturing, overlapping flaps, and the triple-flap method.

(2) Cytoreductive surgery/partial adenomyomectomy, which includes partial removal of the clinically recognizable non-microscopic lesions, avoiding ‘‘functional’’ hysterectomy that can result from complete removal of the lesion with excision of a critical amount of healthy myometrium. This is used in cases of diffuse adenomyosis. This can be achieved by classic excisional techniques for diffuse adenomyosis, transverse H incision technique, wedge resection of the uterine wall, asymmetric dissection of the uterus, or laparoscopically assisted vaginal excision.

(3) Non-excisional techniques, where the removal of adenomyotic tissue is not included, such as laparoscopic uterine artery ligation, hysteroscopic non-excisional techniques, HIFU, RFA, MWA, and balloon thermoablation for diffuse adenomyosis.

The final analysis included nine studies for complete excision, three studies for partial excision, and two for non-excisional techniques. The results showed that partial excision of adenomyosis versus complete excision of adenomyosis did not appear to be statistically significantly different regarding conception rates (46.8% vs. 60.5%), delivery rate (73.3% vs. 83.2%), and miscarriage rate (26.7% vs. 16.9%). They concluded that, although, after complete excision of adenomyosis, there was an increasing trend in fertility, more data are needed to elicit safe results for clinical practice [16].

Similarly, a systematic review by Younes and Tulandi investigated fertility outcomes after partial and complete excision of adenomyosis. However, they used the term “extensive adenomyosis” to describe complete excision of diffuse cases due to the technical infeasibility of performing complete excision in diffuse adenomyosis. Results from 11 studies showed better outcomes after complete excision, with a pregnancy rate of up to 100% compared to 50% in incomplete excision [53].

### 3.5. Other Surgical Approaches

Other surgical techniques have been proposed for the treatment of adenomyosis, including radiofrequency (RF) ablation, microwave ablation (MWA), high-intensity focused ultrasound (HIFU), and hysteroscopic resection. However, the available data focusing on women trying to conceive after these techniques are limited. Radiofrequency (RF) ablation serves as an invasive thermal energy source. An electrode is introduced under ultrasound guidance and electrode(s) are transcervically, laparoscopically, or percutaneously inserted into the lesions, with the generated heat acting directly on the target tissues [54]. RF ablation stands as an established treatment for fibroids due to its minimal invasion, favorable efficacy, and low complication rates. However, there are limited data on microwave (MW) and RF ablation for adenomyosis [54,55].

A meta-analysis of seven articles reviewed the outcomes after radiofrequency. Fertility and reproductive outcomes were reported in only two articles, totaling 41 pregnancies from 31 patients. The overall clinical pregnancy rate was 35.8% and reached 50% after exclusion of women who did not attempt to conceive or gave up attempting pregnancy. The total delivery rate was 66.7%, with the majority delivered via cesarean section. There were three preterm births and no uterine ruptures [55]. RF can be performed alone or in combination with medical therapy, such as GnRH agonist 1–6 months prior to RFA, to decrease the size of pathology in cases of diffuse adenomyosis or LNG-IUD after RFA for symptomatic relief [56,57]. A recent study compared laparoscopic surgery to RF with and without GnRH agonist for 3 months after surgery, with 35 patients in each of the four groups. Interestingly, RF combined with GnRH agonist resulted in the highest pregnancy rate (74.29%) compared to RF without GnRH (45.71%) and laparoscopy with GnRH (44.00%) and without (37.14%) GnRH agonist [58].

HIFU generates ultrasound waves that travel through the body, converging at a focal point. It is delivered by a piezoelectric transducer with a fixed aperture and focal length, guided by MRI or ultrasound. The ultrasound energy absorbed by the tissues is converted to heat, causing coagulative necrosis [59]. This heat-induced cellular collapse results from the loss of a subset of proteins vital for cellular functions [60]. Additionally, the cavitation effect and radiation force also contribute to the ablative effect of HIFU. Despite being the most widely used compared to RFA or MWA, its definitive role in treating adenomyosis in patients desiring fertility is undetermined [61].

A recent meta-analysis, including 10 studies (4 in English and 6 in Chinese), reported fertility results of a total of 557 patients resulting in 287 pregnancies, 177 live births, and 66 spontaneous miscarriages. The follow-up duration ranged from 3 to 60 months. The pooled pregnancy rate (PR) for adenomyosis patients after HIFU treatment was 53.4%, and the live birth rate (LBR) was 35.2%, with a considerable amount of heterogeneity across the studies. Based on six studies reporting the mode of conception, the pooled natural pregnancy rate was 40.3%, with a pooled miscarriage rate of 7.8%. No instances of uterine rupture were reported [61]. It is important to note that most studies were published in specialty journals unrelated to obstetrics and gynecology, with only two articles published in mainstream obstetrics and gynecology journals. Additionally, four Chinese articles had affiliations with HIFU research institutions or the HIFU company, raising potential conflicts of interest. Univariate meta-regression analysis showed that study type (single-arm vs. others) and publication journals (mainstream OB/GYN journal vs. others) significantly contributed to the heterogeneity in the pooled pregnancy rate [61].

Hysteroscopic adenomyomectomy is a viable treatment option for adenomyosis, particularly in cases of superficial disease. The surgical objective remains consistent with other techniques: complete removal of adenomyotic tissue while preserving healthy myometrium and endometrium. However, the absence of a clear plane poses a challenge during hysteroscopy, particularly in relation to larger disease extent [62]. Various techniques are available for treating adenomyomas, with selection contingent upon myometrial location (submucous or intramural), extent (focal or diffuse), and adenomyoma size (<1.5 cm or ≥1.5 cm), and these techniques have been described mostly on patients with cystic adenomyosis [62,63]. For submucous focal and small adenomyomas, hysteroscopic scissors resection is favored. Conversely, intramural focal, large adenomyomas, or superficial diffuse disease typically warrant ablative techniques such as resectoscopic resection or rollerball ablation [62,64]. In cases of localized intramural disease, a spirotome can create a channel to reach the affected tissue, allowing for resection using the aforementioned ablation techniques [63].

Hysteroscopic adenomyomectomy demonstrates notable efficacy in alleviating dysmenorrhea and menorrhagia, as evidenced by Xia et al.’s study involving 51 women undergoing resectoscopic resection, with a 2-year follow-up. The remission rate for menorrhagia was 100% at 3 months, 88% at 6–18 months, and 85% at 24 months, accompanied by a VAS score decrease of 95% at 3 months, 93% at 6 months, 88% at 12 months, and 87% at 18 and 24 months, which shows the effectivity of hysteroscopic adenomyomectomy for symptomatic improvement [65]. However, data regarding fertility and pregnancy outcomes post-hysteroscopic adenomyomectomy remain inconclusive, and more studies are needed to evaluate its effects.

## 4. Discussion

Adenomyosis diagnosis historically relied mainly on histopathological confirmation post-hysterectomy. However, advancements in imaging modalities now enable accurate diagnosis using MRI and ultrasound (US). Multiple imaging classifications have been proposed in the last decade [15]. A recent consensus of the widely used MUSA ultrasound criteria, proposed by an expert group, aims to clearly define the US features of adenomyosis [66]. Different grades and types are categorized based on the number and size of foci, myometrial location (outer/inner), and the number of uterine walls involved [17,18].

A few studies have explored the relationship between the described diagnostic features of adenomyosis and clinical outcomes. Bourdon et al. investigated the clinical profiles of different types of adenomyosis, revealing a higher prevalence of external adenomyosis with endometriosis, while internal adenomyosis is more associated with previous uterine surgeries, with no difference in pain scores [67]. Adenomyosis is known to negatively impact fertility and decrease success rates in assisted reproductive technology (ART). A recent study investigated various MRI features and their relation to ART outcomes, showing that the presence of T2 high-signal intensity myometrial spots independently correlates with a decrease in the cumulative chance of live birth [68].

The definitive treatment for adenomyosis is hysterectomy. However, in patients desiring to maintain fertility, such definitive treatment is not feasible. Uterus-sparing surgeries have become a vital option in these clinical scenarios, particularly after multiple ART failures and/or recurrent miscarriages. Reviewing the literature on this topic revealed multiple deficiencies in the available data, including study heterogeneity with numerous variables such as disease extent, concomitant pathologies like endometriosis or fibroids, patient age, and surgical technique. This heterogeneity results in a wide range of pregnancy rates (30–74%) after conservative surgery, making it challenging to draw conclusive answers regarding the benefits of these excisional surgeries. Additionally, combining surgery with post-surgical medical management has shown improved outcomes compared to surgery alone. Furthermore, ART results have indicated higher pregnancy rates, highlighting potential additional benefits.

Despite the clear benefits shown by available data regarding uterus-sparing surgeries, all studies lack crucial information regarding the mention of adenomyosis subtypes, instead grouping the pathology into two major categories (focal/diffuse). With growing efforts to link proposed subgroups to clinical symptoms and fertility impact, it is crucial to demonstrate the impact of these surgeries on different proposed adenomyosis subgroups.

The strength of this review lies in its comprehensive and updated overview of the available literature on both fertility and pregnancy outcomes after uterus-sparing surgeries in adenomyosis. We investigated surgical, combined surgical, and medical approaches in patients desiring pregnancy and highlighted pregnancy outcomes. Additionally, we shed light on non-excisional techniques, which showed comparable and promising results to excisional approaches. However, a limitation of our study is that it lacked pooled statistical results due to its narrative nature. Nevertheless, most available literature comprises observational studies with high heterogeneity, potentially limiting the generation of high-quality guidance. Furthermore, including more than one database may yield more relevant studies.

### Consideration and Future Directions

Future well-designed comparative studies are necessary, along with efforts to devise a universal classification system applicable to studies focusing on fertility outcomes. This system should accurately delineate the size and location of foci, the extent of adenomyosis, the number of uterine walls involved, the effect on the junctional zone (JZ), and the presence of concomitant pathology. Consistent utilization of a detailed classification will help bridge the knowledge gap between symptom severity and the potential benefits of uterus-sparing surgeries based on the type of adenomyosis. Addressing these questions is crucial: Does uterus-sparing surgery improve fertility outcomes for adenomyosis? Do uterus-sparing procedures benefit all patients across all adenomyosis subgroups? Is one surgical technique superior to another in terms of alleviating symptoms, enhancing fertility outcomes and minimizing complications in future pregnancies? Answering these questions will ultimately empower physicians to implement evidence-based, patient-tailored management plans according to adenomyosis subtypes, leading to enhanced outcomes.

## 5. Conclusions

Various treatment options are available for adenomyosis, ranging from medical to surgical interventions, with hysterectomy being the definitive treatment. Despite these abundant options, infertility patients are left with limited choices that can effectively manage their symptoms while also improving fertility outcomes. Available data suggests that uterine-preserving surgeries, with or without medical treatment, may positively impact fertility and pregnancy outcomes. However, due to the lack of high-quality evidence, further well-designed studies with standardized classification systems, surgical techniques, and additional medical therapies are needed to better understand the optimal approach for patient care while mitigating associated risks, such as uterine rupture and placenta accreta spectrum in subsequent pregnancies. HIFU, hysteroscopy, and radiofrequency ablation represent less invasive options, but few studies are available to draw conclusive results or establish a consensus on their efficacy in enhancing fertility in cases of adenomyosis. Therefore, further trials are necessary to determine the optimal fertility-preserving treatment for adenomyosis.

## Figures and Tables

**Table 4:** Summary of systematic reviews and meta-analysis.

Systematic Reviews and Meta-Analysis of Fertility Outcomes after Adenomyosis Uterus-Sparing Surgery	
Tan et al., (2018) [34].	- Included 16 studies. - Primary purpose to evaluate reproductive outcomes after conservative surgery for both focal and diffuse AD, specifically in patients desiring fertility. - Higher mean pregnancy rates (PRs) and live birth rates were observed in focal cases compared to diffuse cases (52.7% vs. 34.1%) and (43.5% vs. 25%), although significant heterogeneity between studies limits the overall validity of such a comparison. - Comparable PRs can be achieved through both natural conception and assisted reproductive technology (ART) after fertility-conserving surgery for focal and diffuse AD. 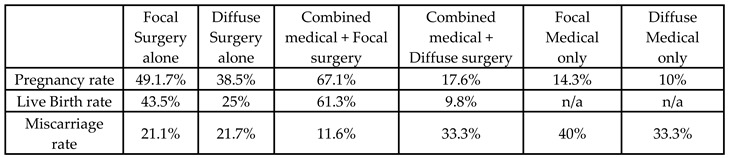
Rocha et al., 2018 [37].	- Included 16 articles to assess the reproductive outcomes of conservative treatments for patients with adenomyosis-associated infertility. - Six studies focusing on surgical treatment; overall pooled clinical pregnancy rate after surgical resection was 38.8%, while the overall clinical pregnancy rate was notably low at 18.2% when considering spontaneous pregnancies. - After GnRH-a were utilized for 24 weeks post-surgery, the pooled spontaneous pregnancy rate was significantly higher compared to not using adjuvant GnRH-a (40.7% vs. 15.0%). - No significant difference observed between the pooled results with or without GnRH-a post-adenomyomectomy concerning pregnancy rate, live birth rate, in vitro fertilization (IVF) pregnancy rate, or miscarriage rate. - Ten studies focusing on ART had an overall clinical pregnancy rate of 36.1%, an overall miscarriage rate of 25.9%, and an overall live birth/ongoing pregnancy rate of 29.9%. - Long vs. short stimulation protocols of ART showed a higher pooled clinical pregnancy rate (43.3% vs. 31.8%, respectively), a higher live birth/ongoing pregnancy rate (43% vs. 23.1%), and a lower frequency of miscarriage (18.5% vs. 31.1%).
Jiang et al. (2023) [52].	- Included 13 studies in divided into 2 categories: (1) complete/incomplete excision of AD and (2) nonexcisional techniques, such as HIFU, UAE, MWA, and RFA. - No statistical differences in the pregnancy rates between the excisional group (40%) and the non-excisional group (51%), with high heterogeneity in the excisional group. - No statistical difference between the excisional and non-excisional groups in the rates of miscarriage - (21% vs. 22%) or live births (70% vs. 71%) simultaneously; no heterogeneity detected in both groups. - The excisional group had higher PR via ART (41%) compared to the natural approach (28%).
Grimbizis et al. (2014) [16]	- Included 12 studies in the final analysis. - Data were grouped into three proposed categories according to the extent of excision: (1) Complete excision of adenomyosis for localized and diffuse AD. (2) Cytoreductive surgery/partial adenomyomectomy for diffuse AD. (3) Non-excisional techniques. - Results showed that partial excision of adenomyosis versus complete excision of adenomyosis did not appear to be statistically significantly different regarding conception rates (46.8% vs. 60.5%), delivery rate (73.3% vs. 83.2%), and miscarriage rate (26.7% vs. 16.9%).
Younes and Tulandi (2018) [53]	- Included 11 studies that evaluated fertility outcomes. - Compared partial and complete excision of adenomyosis. The term “extensive adenomyosis” was used to describe complete excision of diffuse cases. - The pregnancy rate was up to 100% after complete excision compared to up to 50% in incomplete excision.

## Data Availability

All data generated or analyzed during this study are included in this published article.

## References

[1] Mishra I., Melo P., Easter C., Sephton V., Dhillon-Smith R., Coomarasamy A. (2023). Prevalence of adenomyosis in women with subfertility: Systematic review and meta-analysis. Ultrasound Obstet. Gynecol..

[2] Upson K., Missmer S.A. (2020). Epidemiology of Adenomyosis. Semin. Reprod. Med..

[3] Deblaere L., Froyman W., Bosch T.V.D., Van Rompuy A., Kaijser J., Deprest J., Timmerman D. (2019). Juvenile cystic adenomyosis: A case report and review of the literature. Australas. J. Ultrasound Med..

[4] Exacoustos C., Lazzeri L., Martire F.G., Russo C., Martone S., Centini G., Piccione E., Zupi E. (2021). Ultrasound Findings of Adenomyosis in Adolescents: Type and Grade of the Disease. J. Minim. Invasive Gynecol..

[5] Taran F.A., Stewart E.A., Brucker S. (2013). Adenomyosis: Epidemiology, Risk Factors, Clinical Phenotype and Surgical and Interventional Alternatives to Hysterectomy. Geburtshilfe Und Frauenheilkd..

[6] Sharara F.I., Kheil M.H., Feki A., Rahman S., Klebanoff J.S., Ayoubi J.M., Moawad G.N. (2021). Current and Prospective Treatment of Adenomyosis. J. Clin. Med..

[7] Moawad G., Kheil M.H., Ayoubi J.M., Klebanoff J.S., Rahman S., Sharara F.I. (2022). Adenomyosis and infertility. J. Assist. Reprod. Genet..

[8] Tsui K.-H., Lee F.-K., Seow K.-M., Chang W.-C., Wang J.-W., Chen S.-U., Chao H.-T., Yen M.-S., Wang P.-H. (2015). Conservative surgical treatment of adenomyosis to improve fertility: Controversial values, indications, complications, and pregnancy outcomes. Taiwan. J. Obstet. Gynecol..

[9] Li J.-J., Chung J.P.W., Wang S., Li T.-C., Duan H. (2018). The Investigation and Management of Adenomyosis in Women Who Wish to Improve or Preserve Fertility. BioMed Res. Int..

[10] Oliveira M.A.P., Crispi C.P., Brollo L.C., De Wilde R.L. (2017). Surgery in adenomyosis. Arch. Gynecol. Obstet..

[11] Puente J.M., Fabris A., Patel J., Patel A., Cerrillo M., Requena A., Garcia-Velasco J.A. (2016). Adenomyosis in infertile women: Prevalence and the role of 3D ultrasound as a marker of severity of the disease. Reprod. Biol. Endocrinol..

[12] Guo S.-W. (2022). Cracking the enigma of adenomyosis: An update on its pathogenesis and pathophysiology. Reproduction.

[13] Khan K.N., Fujishita A., Mori T. (2022). Pathogenesis of Human Adenomyosis: Current Understanding and Its Association with Infertility. J. Clin. Med..

[14] Vercellini P., Viganò P., Bandini V., Buggio L., Berlanda N., Somigliana E. (2023). Association of endometriosis and adenomyosis with pregnancy and infertility. Fertil. Steril..

[15] Moawad G., Fruscalzo A., Youssef Y., Kheil M., Tawil T., Nehme J., Pirtea P., Guani B., Afaneh H., Ayoubi J.M. (2023). Adenomyosis: An Updated Review on Diagnosis and Classification. J. Clin. Med..

[16] Grimbizis G.F., Mikos T., Tarlatzis B. (2014). Uterus-sparing operative treatment for adenomyosis. Fertil. Steril..

[17] Exacoustos C., Morosetti G., Conway F., Camilli S., Martire F.G., Lazzeri L., Piccione E., Zupi E. (2020). New Sonographic Classification of Adenomyosis: Do Type and Degree of Adenomyosis Correlate to Severity of Symptoms?. J. Minim. Invasive Gynecol..

[18] Bazot M., Daraï E. (2018). Role of transvaginal sonography and magnetic resonance imaging in the diagnosis of uterine adenomyosis. Fertil. Steril..

[19] Fedele L., Bianchi S., Zanotti F., Marchini M., Candiani G.B. (1993). Surgery: Fertility after conservative surgery for adenomyomas. Hum. Reprod..

[20] Fujishita A., Masuzaki H., Khan K.N., Kitajima M., Ishimaru T. (2004). Modified reduction surgery for adenomyosis. A preliminary report of the transverse H incision technique. Gynecol. Obstet. Investig..

[21] Wang P.-H., Liu W.-M., Fuh J.-L., Cheng M.-H., Chao H.-T. (2008). Comparison of surgery alone and combined surgical-medical treatment in the management of symptomatic uterine adenomyoma. Fertil. Steril..

[22] Guy M.M., Ying W.Z., Yan W.X., Hui F.Z., Hai W.X., Ping L.Y., Chun Z.Y., Aurelie K.K., Tao W.Z. (2016). Effectiveness of treatment for infertility using clinical investigation of laparoscopy cytoreductive surgery combined with gestrinone in adenomyosis. Gynecol. Surg..

[23] Kishi Y., Yabuta M., Taniguchi F. (2014). Who will benefit from uterus-sparing surgery in adenomyosis-associated subfertility?. Fertil. Steril..

[24] Kishi Y., Suginami H., Kuramori R., Yabuta M., Suginami R., Taniguchi F. (2012). Four subtypes of adenomyosis assessed by magnetic resonance imaging and their specification. Am. J. Obstet. Gynecol..

[25] Tamura H., Kishi H., Kitade M., Asai-Sato M., Tanaka A., Murakami T., Minegishi T., Sugino N. (2017). Clinical outcomes of infertility treatment for women with adenomyosis in Japan. Reprod. Med. Biol..

[26] Takeuchi H., Kitade M., Kikuchi I., Kumakiri J., Kuroda K., Jinushi M. (2010). Diagnosis, laparoscopic management, and histopathologic findings of juvenile cystic adenomyoma: A review of nine cases. Fertil. Steril..

[27] Acien P., Bataller A., Fernandez F., Acien M.I., Rodriguez J.M., Mayol M.J. (2012). New cases of accessory and cavitated uterine masses (ACUM): A significant cause of severe dysmenorrhea and recurrent pelvic pain in young women. Hum. Reprod..

[28] Boitor-Borza D., Rotar C., Muresan D. (2023). Accessory cavitated uterine mass in a multiparous patient with progressive dysmenorrhea. Am. J. Obstet. Gynecol..

[29] Kwon Y., Roh H.J., Ahn J.W., Lee S., Im K.S. (2014). Conservative adenomyomectomy with transient occlusion of uterine arteries for diffuse uterine adenomyosis. J. Obstet. Gynaecol. Res..

[30] Kwack J., Kwon Y. (2018). Conservative surgery of diffuse adenomyosis with TOUA: Single surgeon experience of one hundred sixteen cases and report of fertility outcomes. Kaohsiung J. Med. Sci..

[31] Saremi A., Bahrami H., Salehian P., Hakak N., Pooladi A. (2014). Treatment of adenomyomectomy in women with severe uterine adenomyosis using a novel technique. Reprod. Biomed. Online.

[32] Fujishita A., Hiraki K., Kitajima M., Matsumoto Y., Satoh H., Masuzaki H. (2010). Shikyusenkinsho to shikyu no onzon-chiryo (Uterine adenomyosis and uterine preservation treatment). J. Obstet. Gynecol. Prac..

[33] Yoon S.H., Lee G.J.B., Cho H.J., Kwon H., Yun B.S., Lee C.H., Park H.S., Roh J.-W. (2023). Clinical efficacy of a novel method of fertility-preserving adenomyomectomy in infertile women with diffuse adenomyosis. Medicine.

[34] Tan J., Moriarty S., Taskin O., Allaire C., Williams C., Yong P., Bedaiwy M.A. (2018). Reproductive Outcomes after Fertility-Sparing Surgery for Focal and Diffuse Adenomyosis: A Systematic Review. J. Minim. Invasive Gynecol..

[35] Osada H., Silber S., Kakinuma T., Nagaishi M., Kato K., Kato O. (2011). Surgical procedure to conserve the uterus for future pregnancy in patients suffering from massive adenomyosis. Reprod. Biomed. Online.

[36] Moawad G., Youssef Y., Fruscalzo A., Faysal H., Kheil M., Pirtea P., Guani B., Ayoubi J.M., Feki A. (2023). The Present and the Future of Medical Therapies for Adenomyosis: A Narrative Review. J. Clin. Med..

[37] Rocha T.P., Andres M.P., Borrelli G.M., Abrão M.S. (2018). Fertility-Sparing Treatment of Adenomyosis in Patients with Infertility: A Systematic Review of Current Options. Reprod. Sci..

[38] Rajuddin R., Jacoeb T.Z. (2006). Management of adenomyosis in infertile women: Comparison between laparotomic resection and administration of aromatase inhibitor (Experience in 55 cases). Med. J. Indones..

[39] Hadisaputra W., Anggraeni T.D. (2006). Laparoscopic resection versus myolysis in the management of symptomatic uterine adenomyosis: Alternatives to conventional treatment. Med. J. Indones..

[40] Al Jama F. (2011). Management of Adenomyosis in Subfertile Women and Pregnancy Outcome. Oman Med. J..

[41] Chang W.-H., Wang K.-C., Lee N.-R., Huang N., Su W.-H., Chao H.-T., Yen M.-S., Fuh J.-L., Wang P.-H. (2013). Reproductive performance of severely symptomatic women with uterine adenomyoma who wanted preservation of the uterus and underwent combined surgical–medical treatment. Taiwan. J. Obstet. Gynecol..

[42] Huang B.-S., Seow K.-M., Tsui K.-H., Huang C.-Y., Lu Y.-F., Wang P.-H. (2012). Fertility outcome of infertile women with adenomyosis treated with the combination of a conservative microsurgical technique and GnRH agonist: Long-term follow-up in a series of nine patients. Taiwan. J. Obstet. Gynecol..

[43] Zhou Y., Shen L., Wang Y., Yang M., Chen Z., Zhang X. (2022). Long-Term Pregnancy Outcomes of Patients with Diffuse Adenomyosis after Double-Flap Adenomyomectomy. J. Clin. Med..

[44] Wang P., Fuh J., Chao H., Liu W., Cheng M., Chao K. (2009). Is the surgical approach beneficial to subfertile women with symptomatic extensive adenomyosis?. J. Obstet. Gynaecol. Res..

[45] Hashimoto A., Iriyama T., Sayama S., Nakayama T., Komatsu A., Miyauchi A., Nishii O., Nagamatsu T., Osuga Y., Fujii T. (2018). Adenomyosis and adverse perinatal outcomes: Increased risk of second trimester miscarriage, preeclampsia, and placental malposition. J. Matern. Neonatal Med..

[46] Kwack J.-Y., Lee S.-J., Kwon Y.-S. (2021). Pregnancy and delivery outcomes in the women who have received adenomyomectomy: Performed by a single surgeon by a uniform surgical technique. Taiwan. J. Obstet. Gynecol..

[47] Sayama S., Iriyama T., Hashimoto A., Suzuki K., Ariyoshi Y., Yano E., Toshimitsu M., Ichinose M., Seyama T., Sone K. (2023). Possible risks and benefits of adenomyomectomy on pregnancy outcomes: A retrospective analysis. AJOG Glob. Rep..

[48] Sugiyama M., Takahashi H., Baba Y., Taneichi A., Suzuki H., Usui R., Takei Y., Ohkuchi A., Fujiwara H., Matsubara S. (2020). Perinatal outcome of pregnancy after adenomyomectomy: Summary of 10 cases with a brief literature review. J. Matern. Neonatal Med..

[49] Otsubo Y., Nishida M., Arai Y., Ichikawa R., Taneichi A., Sakanaka M. (2015). Association of uterine wall thickness with pregnancy outcome following uterine-sparing surgery for diffuse uterine adenomyosis. Aust. N. Zldn. J. Obstet. Gynaecol..

[50] Ono Y., Ota H., Fukushi Y., Tagaya H., Okuda Y., Yoshino O., Yamada H., Hirata S., Wada S. (2023). Effectiveness of Laparoscopic Adenomyomectomy on Perinatal Outcomes. Gynecol. Minim. Invasive Ther..

[51] Nishida M., Takano K., Arai Y., Ozone H., Ichikawa R. (2010). Conservative surgical management for diffuse uterine adenomyosis. Fertil. Steril..

[52] Jiang L., Han Y., Song Z., Li Y. (2023). Pregnancy Outcomes after Uterus-sparing Operative Treatment for Adenomyosis: A Systematic Review and Meta-analysis. J. Minim. Invasive Gynecol..

[53] Younes G., Tulandi T. (2018). Conservative Surgery for Adenomyosis and Results: A Systematic Review. J. Minim. Invasive Gynecol..

[54] Lin X.L., Hai N., Zhang J., Han Z.Y., Yu J., Liu F.Y., Dong X.J., Liang P. (2020). Comparison between microwave ablation and radiofrequency ablation for treating symptomatic uterine adenomyosis. Int. J. Hyperth..

[55] Dedes I., Kolovos G., Arrigo F., Toub D., Vaineau C., Lanz S., Imboden S., Feki A., Mueller M.D. (2023). Radiofrequency Ablation for Adenomyosis. J. Clin. Med..

[56] Nam J. (2019). Pregnancy and symptomatic relief following ultrasound-guided transvaginal radiofrequency ablation in patients with adenomyosis. J. Obstet. Gynaecol. Res..

[57] Hai N., Hou Q., Guo R. (2021). Ultrasound-guided transvaginal radiofrequency ablation combined with levonorgestrel-releasing intrauterine system for symptomatic uterine adenomyosis treatment. Int. J. Hyperth..

[58] Chu Z., Jia L., Dai J., Wu Q., Tian F., Bai S. (2024). Effects of different treatment methods on clinical efficacy and fertility outcomes of patients with adenomyosis. J. Ovarian Res..

[59] Elhelf I.S., Albahar H., Shah U., Oto A., Cressman E., Almekkawy M. (2018). High intensity focused ultrasound: The fundamentals, clinical applications and research trends. Diagn. Interv. Imaging.

[60] Leuenberger P., Ganscha S., Kahraman A., Cappelletti V., Boersema P.J., von Mering C., Claassen M., Picotti P. (2017). Cell-wide analysis of protein thermal unfolding reveals determinants of thermostability. Science.

[61] Chen Y., Lin S., Xie X., Yi J., Liu X., Guo S.-W. (2024). Systematic review and meta-analysis of reproductive outcomes after high-intensity focused ultrasound (HIFU) treatment of adenomyosis. Best Pr. Res. Clin. Obstet. Gynaecol..

[62] Sardo A.D.S., Calagna G., Santangelo F., Zizolfi B., Tanos V., Perino A., De Wilde R.L. (2017). The Role of Hysteroscopy in the Diagnosis and Treatment of Adenomyosis. BioMed Res. Int..

[63] Gordts S., Campo R., Brosens I. (2014). Hysteroscopic diagnosis and excision of myometrial cystic adenomyosis. Gynecol. Surg..

[64] Preutthipan S., Herabutya Y. (2010). Hysteroscopic rollerball endometrial ablation as an alternative treatment for adenomyosis with menorrhagia and/or dysmenorrhea. J. Obstet. Gynaecol. Res..

[65] Xia W., Zhang D., Zhu Q., Zhang H., Yang S., Ma J., Pan H., Tong T., Sun J., Zhang J. (2017). Hysteroscopic excision of symptomatic myometrial adenomyosis: Feasibility and effectiveness. BJOG Int. J. Obstet. Gynaecol..

[66] Harmsen M.J., Bosch T.V.D., de Leeuw R.A., Dueholm M., Exacoustos C., Valentin L., Hehenkamp W.J.K., Groenman F., De Bruyn C., Rasmussen C. (2022). Consensus on revised definitions of Morphological Uterus Sonographic Assessment (MUSA) features of adenomyosis: Results of modified Delphi procedure. Ultrasound Obstet. Gynecol..

[67] Bourdon M., Oliveira J., Marcellin L., Santulli P., Bordonne C., Mantelet L.M., E Millischer A., Bureau G.P., Chapron C. (2020). Adenomyosis of the inner and outer myometrium are associated with different clinical profiles. Hum. Reprod..

[68] Bourdon M., Santulli P., Bordonne C., E Millisher A., Maitrot-Mantelet L., Maignien C., Marcellin L., Melka L., Chapron C. (2022). Presence of adenomyosis at MRI reduces live birth rates in ART cycles for endometriosis. Hum. Reprod..

